# The effects of high-flow nasal cannula on intubation and re-intubation in critically ill patients: a systematic review, meta-analysis and trial sequential analysis

**DOI:** 10.5935/0103-507X.20180070

**Published:** 2018

**Authors:** Rafael Ladeira Rosa Bocchile, Denise Carnieli Cazati, Karina Tavares Timenetsky, Ary Serpa Neto

**Affiliations:** 1 Departamento de Terapia Intensiva, Hospital Israelita Albert Einstein - São Paulo (SP), Brasil.; 2 Departamento de Terapia Intensiva e Laboratório Experimental de Terapia Intensiva e Anestesiologia, Centro Médico Acadêmico, Universidade de Amsterdã - Amsterdã, Holanda.

**Keywords:** Catheters, Oxygen inhalation therapy, Noninvasive ventilation, Intubation, intratracheal

## Abstract

**Objective:**

To evaluate the efficacy of high-flow nasal cannula in the prevention of intubation and re-intubation in critically ill patients compared to conventional oxygen therapy or noninvasive ventilation.

**Methods:**

This systematic review was performed through an electronic database search of articles published from 1966 to April 2018. The primary outcome was the need for intubation or re-intubation. The secondary outcomes were therapy escalation, mortality at the longest follow-up, hospital mortality and the need for noninvasive ventilation.

**Results:**

Seventeen studies involving 3,978 patients were included. There was no reduction in the need for intubation or re-intubation with high-flow nasal cannula (OR 0.72; 95%CI 0.52 - 1.01; p = 0.056). There was no difference in the need for therapy escalation (OR 0.80, 95% CI 0.59 - 1.08, p = 0.144), mortality at the longest follow-up (OR 0.94; 95%CI 0.70 - 1.25; p = 0.667), hospital mortality (OR 0.84; 95%CI 0.56 - 1.26; p = 0.391) or noninvasive ventilation (OR 0.64, 95%CI 0.39 - 1.05, p = 0.075). In the trial sequential analysis, the number of events included was lower than the optimal information size with a global type I error > 0.05.

**Conclusion:**

In the present study and setting, high-flow nasal cannula was not associated with a reduction of the need for intubation or re-intubation in critically ill patients.

## INTRODUCTION

An oxygen supply with flows higher than 6L/minute is considered a high-flow therapy; however, under standard care, this supply is generally not heated or humidified and can reach a maximum flow of 15L/min.^(1)^ The use of the a high-flow nasal cannula (HFNC) allows flow rates up to 60L/min because of the use of a heater and humidifier.^(2)^ This heated air provides a relative humidity of 100%, which improves the work of the mucociliary epithelium and provides greater comfort to the patient.

The following physiological effects of the HFNC should be highlighted: 1) reduction of anatomical dead space; 2) decrease in airway resistance; 3) increase in lung compliance; 4) improvement in bronchial hygiene; and 5) maintenance of a certain level of positive pressure at the end of expiration (approximately 3 - 6cmH_2_O).^(1-6)^Clinically, these physiological effects translate into decreased respiratory work during breathing and improvement of hypoxemia.^(1-6)^Additionally, some of its advantages include the comfort reported by the patient compared to conventional oxygen therapy or non-invasive ventilation (NIV) and the decrease in the sensation of dyspnea that can be explained by high inspiratory flow.^(4)^

Recent studies suggest the application of the HFNC primarily for cases of hypoxemic respiratory failure, post-extubation of medical and surgical patients, when the use of NIV is contraindicated or when there is no adaptation to its use, and in special situations, such as palliative care and relief of dyspnea.^(1-4)^ In general, the HFNC can also be used as a safe alternative in cases of hypoxemic respiratory failure and to avoid intubation in critically ill patients compared to conventional oxygen therapy or NIV.^(6-8)^

We performed a meta-analysis to assess the effects of the HFNC on the need for intubation or re-intubation in adult critically ill patients compared to conventional oxygen therapy or NIV. We hypothesized that the use of HFNC is associated with a decreased need for intubation or re-intubation.

## METHODS

### Search strategy

This systematic review was performed through electronic searches in the PubMed, Web of Science, Cumulative Index of Nursing and Allied Health® (CINAHL®) and CENTRAL databases from 1966 to April 2018 by two independent and blinded investigators. A search strategy incorporating keywords and utilizing Medical Subject Headings (MeSH) was used: ("high flow nasal oxygen" OR "high flow nasal cannula" OR "HFNO" OR "HFNC" OR "high flow oxygen"). All articles returned for this query were scanned for relevance by title and abstract. For potentially relevant articles, the full text was obtained for review; the references of these articles and related reviews and meta-analyses were inspected, and potentially relevant titles were hand searched. No further limitations were set on the query.

### Selection of the studies

The following inclusion criteria were used: randomized clinical trials; adult patient population (age ≥ 18 years); and compared the use of HNFC 4) to NIV or to conventional oxygen therapy (nasal cannula or facial mask). Crossover studies, or studies that focused on the use of HFNC during procedures or during palliative care, were excluded.

### Data extraction and quality assessment

Two independent investigators conducted the electronic search and extracted the data into a database developed for the study. A third investigator was called for discussion if there was disagreement between the first two investigators. To evaluate the risk of bias in the studies, the Cochrane Risk of Bias Tool was used. Studies indicated as "low risk of bias" were studies with a low risk of bias in all domains.

### Outcomes

The primary outcome was the need for intubation or re-intubation during the follow-up. The following secondary outcomes were evaluated: (1) need for therapy escalation (defined as the need for NIV or invasive ventilation in the HFNC group, the need for invasive ventilation in the NIV group and the need for NIV, HFNC or invasive ventilation in the group with conventional oxygen therapy); (2) mortality at longest follow-up (defined as the mortality reported at the last follow-up); (3) hospital mortality; and (4) the need for NIV (assessed in the HFNC and conventional oxygen therapy groups).

### Analysis plan

The treatment group in the present study was the group treated with the HFNC whereas the control group was the group treated with NIV or conventional oxygen therapy (independent of the interface used to offer the therapy). All analyses were stratified according to the type of primary outcome: intubation or re-intubation. In relation to the controls, the following groups were considered: NIV or conventional oxygen therapy. The main findings are stratified according to the type of outcome reported by the studies (intubation *versus* re-intubation).

### Statistical analysis

All studies included in the systematic review were analyzed in the meta-analysis. For the dichotomous data, the odds ratio (OR) was calculated for the individual studies using a random effects model according to DerSimonian-Laird, and the results were plotted using forest plots. The heterogeneity was measured by I^2^, which describes the total percentage of variation among the studies that is due to heterogeneity rather than chance. I^2^ was calculated according to the following formula: I^2^ = 100% x (Q - df) / Q, where Q is the Cochrane heterogeneity statistic. The results of 0% represent no heterogeneity whereas higher values represent higher heterogeneity.

A subgroup analysis was performed by considering the type of control used (NIV *versus* conventional oxygen therapy). Additionally, the *leave-one-out* method was used to evaluate the validity and consistency of the results of the primary outcome. In addition, a sensitivity analysis according to the indication of the HFNC (post-extubation in surgical patients, post-extubation in clinical patients, respiratory failure in surgical patients and respiratory failure in clinical patients) was performed. The Grading of Recommendations, Assessment, Development and Evaluations (GRADE) method was used to test and report the quality of the evidence.

Because the event size necessary for a very precise meta-analysis is at least as large as that for a single optimally powered randomized controlled trial, the optimal event size requirement for this meta-analysis was calculated based on an intubation rate of 20% in the control group, a relative risk reduction of 25%, 90% power, and a type I error of 5%. The relative risk reduction of 25% was chosen to have adequate power to detect even a small but clinically important effect. Thus, the inclusion of at least 1,262 events was necessary. A formal trial sequential analysis (TSA; TSA software version 0.9 Beta; Copenhagen Trial Unit, Copenhagen, Denmark) was performed using the optimal event size to help to construct sequential monitoring boundaries for the meta-analysis. The boundaries were established to limit the global type I error to 5%. As a sensitivity assessment, a TSA considering a stricter type I error of 1% was conducted since this more conservative approach may be appropriate for a meta-analysis of small trials. As an additional sensitivity analysis, two independent TSAs were performed according to the indication of the HFNC (post-extubation *versus* hypoxemic respiratory failure).

All analyses were performed with Review Manager v. 5.1.1 and R v.3.4.2 (R Foundation for Statistical Computing, Vienna, Austria). For all analyses, p values < 0.05 were considered significant.

## RESULTS

### Study identification

**Figure 1:**
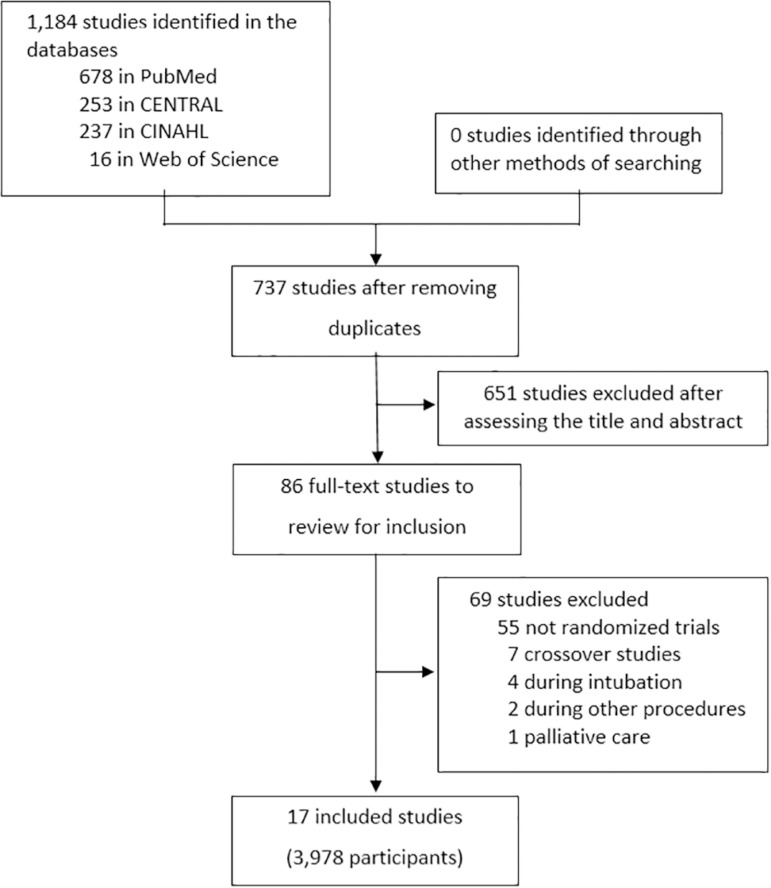
Study flowchart.

The initial search yielded 1,184 studies (678 from PubMed, 16 from Web of Science, 237 from CINAHL and 253 from CENTRAL) (Figure 1). After removing duplicates, the abstracts of 737 studies were evaluated, and 651 studies were excluded. Subsequently, the full text of the remaining 86 studies was analyzed. Sixty-nine were excluded for the following reasons: not randomized clinical trials (*n* = 55); crossover design (n = 7); studies performed during orotracheal intubation (n = 4); studies performed during other procedures (n = 2); and studies in palliative care (n = 1). Finally, 17 studies (3,978 patients) were included in the systematic review (Table 1).^(5-21)^

CINAHL - Cumulative Index of Nursing and Allied Health.

**Table 1:** Characteristics of the included studies

Studies	Multicentric	Control group	Number of patients		Primary outcome
			HFNC group	Control group	
Post-extubation in clinical patients					
Hernandéz^(8)^	Yes	NIV	290	314	Re-intubation in 72 hours
Fernandez^(19)^	Yes	Oxygen	78	77	Respiratory failure in 72 hours
Hernandéz^(20)^	Yes	Oxygen	264	263	Re-intubation in 72 hours
Maggiore^(21)^	Yes	Oxygen	53	52	PaO_2_ / FiO_2_ after 24 hours
Post-extubation in surgical patients					
Brainard^(11)^	No	Oxygen	18	26	Pulmonary complications
Ansari^(15)^	No	Oxygen	28	31	6 m walking test
Corley^(16)^	No	Oxygen	81	74	Atelectasis on chest radiography
Futier^(17)^	Yes	Oxygen	108	112	Hypoxemia
Parke^(18)^	No	Oxygen	169	171	SpO_2_ / FiO_2_ on third day
Hypoxemic respiratory failure in clinical patients					
Frat^(6)^	Yes	NIV/Oxygen	106	94 / 110	Need for MV in 28 days
Rittayamai^(7)^	No	Oxygen	20	20	Dyspnea levels
Azevedo^(9)^	No	NIV	14	16	Need for intubation
Bell^(10)^	Yes	Oxygen	48	52	Reduction in RR
Parke^(12)^	No	Oxygen	29	27	Not specified
Jones^(13)^	No	Oxygen	165	138	Need for NIV or MV
Lemiale^(14)^	Yes	Oxygen	52	48	Need for NIV or MV
Hypoxemic respiratory failure in surgical patients					
Stéphan et al.^(5)^	Yes	NIV	414	416	Treatment failure

HFNC - high-flow nasal cannula; NIV- noninvasive ventilation; PaO_2_ - partial pressure of oxygen; FiO_2_ - inspired fraction of oxygen; SpO_2_ - pulse oximetry; MV- mechanical ventilation; RR - respiratory rate.

### Study characteristics

**Figure 2:**
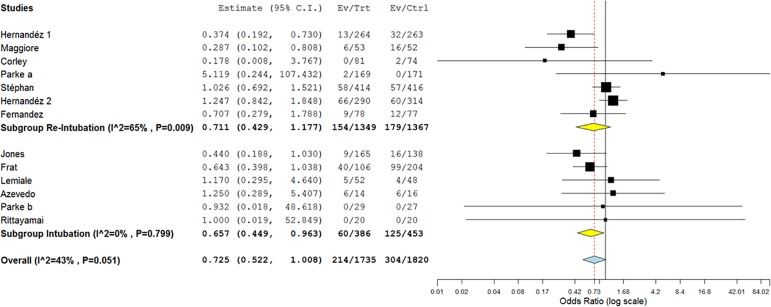
Forest plot comparing the effects of high-flow nasal cannula with the control group for the primary outcome (need for intubation or re-intubation).

The study characteristics are reported in table 1. Most of the studies were multicentric (53%) and used conventional oxygen therapy in the control group (76.5%). The number of patients in each study arm ranged from 14 to 416 participants, and the mean age of the participants was 63.9 ± 5.1 years. The primary outcomes varied according to the studies evaluated. The risk of bias in the studies is reported in figures 1S and 2S (Supplementary material). Most of the studies presented a low risk of selection bias. By contrast, none of the studies were able to blind the participants or the team because of the nature of the intervention, and only two studies blinded the evaluation of the outcomes. For the other components assessed, most of the studies had a low risk of bias.

### Primary outcome

**Figure 3:**
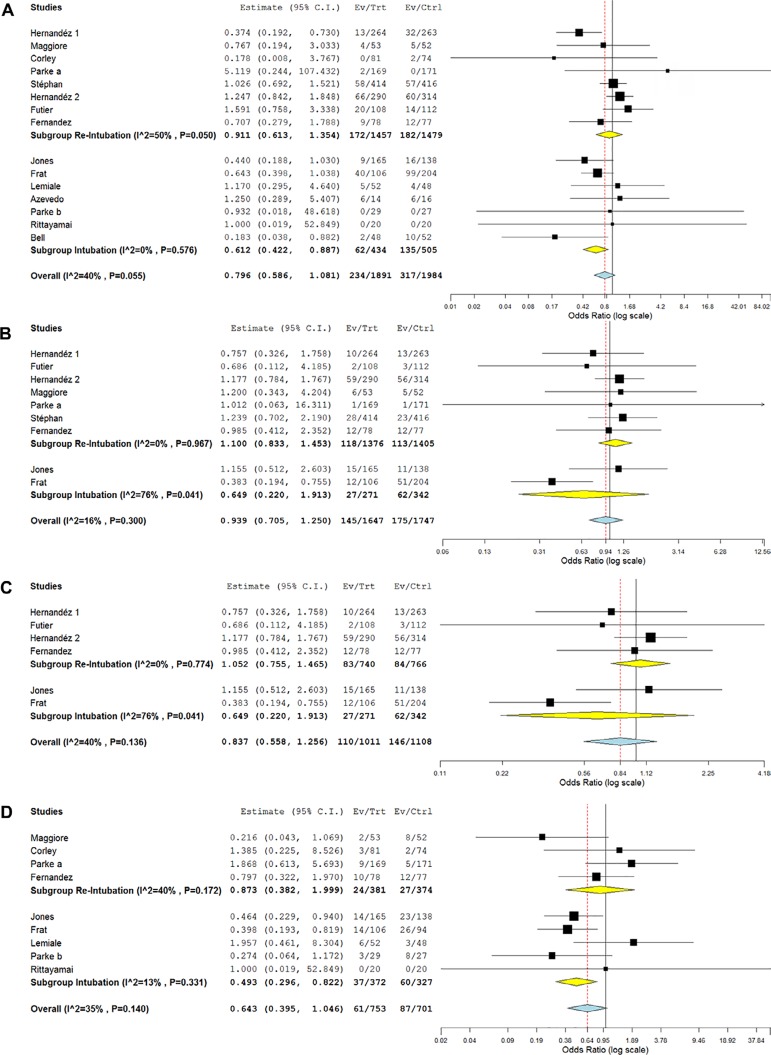
Forest plot comparing the effects of high-flow nasal cannula with the control group for: (A) need for therapy escalation; (B) mortality at the longest follow-up; (C) hospital mortality; and (D) need for noninvasive ventilation.

Thirteen studies assessed the need for intubation or re-intubation. Two hundred and fourteen of the 1,735 patients in the HFNC group and 304 of the 1,820 patients in the control group were intubated or re-intubated during follow-up (OR 0.72; 95%CI 0.52 - 1.01; p = 0.056) (Figure 2). There was a reduction in the need for intubation (OR 0.66; 95%CI 0.45 - 0.96; p = 0.031) but not the need for re-intubation (OR 0.71; 95%CI 0.43 - 1.18; p = 0.185). Mild heterogeneity was found in the analysis (I^2^ = 43%; p = 0.051), predominantly in the re-intubation subgroup (I^2^ = 65%; p = 0.009 *versus* I^2^ = 0%; p = 0.799 in the intubation group) (Figure 2). The leave-one-out analysis confirmed the consistency of the findings as shown in figure 3S (Supplementary material).

95%CI - 95% confidence interval; Ev - events; Trt - treatment; Ctrl - control.

### Secondary outcomes

There was no difference in the need for therapy escalation between the groups (OR 0.80; 95%CI 0.59 - 1.08; p = 0.144). However, in the subgroup of patients in which the need for intubation was assessed as the primary outcome, there was a reduction in the need for therapy escalation (OR 0.61; 95%CI 0.42 - 0.89; p = 0.010). The heterogeneity found in the analysis was also mild (I^2^ = 40%; p = 0.055) and predominantly in the re-intubation subgroup (I^2^ = 50%; p = 0.050 *versus* I^2^ = 0%; p = 0.576 in the intubation group) (Figure 3A). Furthermore, no difference in mortality at the longest follow-up was found (OR 0.94; 95%CI 0.70 - 1.25; p = 0.667), and this was consistent in the two subgroups analyzed. No heterogeneity was found in the analysis (I^2^ = 16%; p = 0.300) (Figure 3B). No differences were found for hospital mortality (OR 0.84; 95%CI 0.56 - 1.26; p = 0.391), independent of the subgroup analyzed. There was moderate heterogeneity in the analysis (I^2^ = 40%; p = 0.136), mainly in the intubation subgroup (I^2^ = 76%; p = 0.041) (Figure 3C). Finally, there was no difference in the need for NIV between the groups (OR 0.64; 95%CI 0.39 - 1.05; p = 0.075); however, in the subgroup of patients in whom the need for intubation was assessed, there was a reduction in the need for the use of NIV (OR 0.49; 95%CI 0.30 - 0.82; p = 0.007). The heterogeneity found in the analysis was also mild (I^2^ = 35%; p = 0.140) and predominantly in the re-intubation subgroup (I^2^ = 40%; p = 0.172 *versus* I^2^ = 13%; p = 0.331 in the intubation group) (Figure 3D).

95%CI - 95% confidence interval; Ev - events; Trt - treatment; Ctrl - control.

### Subgroup analysis

The use of HFNC was associated with a reduced need of intubation only when compared to conventional oxygen therapy (OR 0.54; 95%CI 0.39 - 0.74) but not compared to NIV (OR 0.98; 95%CI 0.70 - 135; p for interaction = 0.010), similar to the findings for therapy escalation (OR 0.66; 95%CI 0.45 - 0.97 compared to conventional oxygen therapy and OR 0.98; 95%CI 0.70 - 1.35 compared to NIV; p for interaction = 0.045) (Table 1S - Supplementary material). No other interaction between the effect of HFNC and the control used was found.

**Figure 4:**
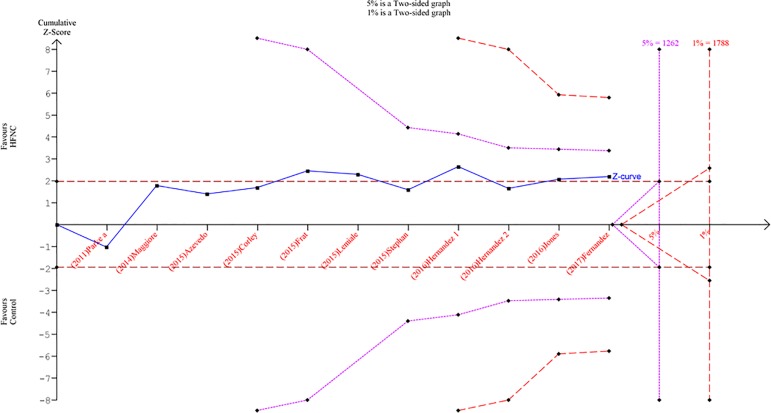
Trial sequential analysis assessing the effect of high-flow nasal cannula in the primary outcome. The cumulative meta-analysis with 518 events (blue line) did not cross the efficacy boundary for the primary outcome (global type I error > 5%; purple line). The same was found when a more conservative boundary was used (red line).

The use of HFNC was associated with a reduced incidence of the primary outcome only in the subgroup that used HFNC due to hypoxemic respiratory failure in clinical patients (OR 0.66; 95%CI 0.45 0.96; p = 0.031) (Figure 4S - Supplementary material).

### Quality of evidence and trial sequential analysis

Based on GRADE, the quality of the evidence is shown in table 2S (Supplementary material). For all outcomes, the quality of evidence was assessed as moderate. A total of 518 events were assessed, which was lower than the estimated optimal event size (1,262 events), and the TSA indicated a global type I error > 5% for the meta-analysis result (Figure 4). The same finding persisted when using an overall type I error limit of 1% and when stratifying according to the indication (Figures 5S and 6S - Supplementary material).

HFNC - high-flow nasal cannula.

## DISCUSSION

The present study aimed to evaluate the effect of HFNC on the prevention of orotracheal intubation or re-intubation in critically ill patients compared to conventional oxygen therapy or NIV. In this group of patients, the use of HFNC reduced the need for intubation but not for re-intubation. Moreover, there was no difference in the need of therapy escalation, mortality at the longest follow-up or hospital mortality between the groups. A secondary analysis, in relation to the type of control used, showed the reduction of intubation with HFNC only when compared with conventional oxygen therapy. The TSA did not achieve the boundaries for efficacy.

Recent studies have examined the use of an HFNC in patients with acute hypoxemic respiratory failure by observing its physiological effects. One study showed a decrease in respiratory work during breathing, with improvements in oxygenation, increases in lung volumes and compliance, and a reduction of carbon dioxide (CO_2_) levels due to the reduction of anatomical dead space and increase of pulmonary ventilation with the use of HFNC.^(22)^In patients with hypoxemic respiratory failure after extubation, the use of HFNC is associated with a decrease in the re-intubation rate, particularly when compared to conventional oxygen therapy. Therefore, the use of HFNC may be a safe alternative in the control of post-extubation respiratory failure and in situations where NIV is contraindicated or not tolerated.^(23^

A recent meta-analysis reported a decrease in the rate of intubation with the HFNC compared to conventional oxygen therapy; however, the rate was similar compared to NIV.^(24)^ Other explanations for the success of the HFNC in this situation might be the adequacy of the minute ventilation and the maintenance of the constant oxygenation by the high nasal flow, which reduces the respiratory work of breathing, improves the abdominal thoracic synchrony and avoids intubation in patients with acute respiratory failure. Another point raised by the study was the decrease of CO_2_ levels and the decrease of anatomical dead space, which may have contributed to the reduction in the rate of intubation compared to conventional oxygen therapy. However, there was no decrease in ICU mortality with HFNC compared to the control.^(24)^ In another published meta-analysis, a decrease in the rate of intubation and in the escalation of respiratory support was reported with the use of the HFNC. Regarding mortality, there was no significant difference between the group that used HFNC and the group managed with NIV or conventional oxygen therapy.^(25)^ Finally, Lin et al. confirmed the findings of the reduction in intubation rate with the use of the HFNC in patients with hypoxemic respiratory failure in comparison to controls in a meta-analysis.^(26)^ In general, these meta-analyses considered fewer studies and fewer conditions of use for the HFNC than the meta-analysis presented in this study.

Among other relevant aspects that differentiate this study from other meta-analyses is the analysis of the results by subgroups, in which the outcomes are compared to the type of control; the use of the "leave-one-out" method to evaluate the consistency of the results; and the use of GRADE to report the quality of the evidence included in this meta-analysis.

The results of this meta-analysis should be interpreted within the context of the included studies since systematic reviews are subject to the overall quality of the studies and publication biases may occur. Still, most of the studies present some risk of bias and were single center, which reduces the external validity of the findings. The presence of heterogeneity in some analyses and the weight of some studies in some evaluations may have influenced the present findings. The fact that most of the outcomes were reported only in some studies, and not in all included studies, is another limitation. In fact, unreported outcomes may lead to overestimation of the effects in a meta-analysis.^(27)^ Furthermore, funnel plots were not used to evaluate the publication bias of the analyses. In general, in situations with some degree of heterogeneity, as in the included analyses, funnel plots add little information.^(28)^ Methods such as Egger's regression or Begg's test also suffer from low power in situations where few studies are included, with assessments suggesting that at least 30 studies are required to yield adequate power for these methods.^(28,29)^

## CONCLUSION

In the present systematic review and meta-analysis, high-flow nasal cannula was not associated with a reduction in the need for intubation or re-intubation in critically ill patients. However, the use of high-flow nasal cannula was associated with a reduction in the need for intubation compared to conventional oxygen therapy. Finally, as suggested by the results of the trial sequential analysis, the present meta-analysis is underpowered to drawn definitive conclusions.

## References

[1] Wattier BA, Ward JJ (2011). High-flow nasal cannula oxygen in critically ill adults do the
nose or lungs know there's a difference?. Respir Care.

[2] Nishimura M (2016). High-flow nasal cannula oxygen therapy in adults physiological
benefits, indication, clinical benefits, and adverse effects. Respir Care.

[3] Dres M, Demoule A (2017). What every intensivist should know about using high-flow nasal
oxygen for critically ill patients. Rev Bras Ter Intensiva.

[4] Schwabbauer N, Berg B, Blumenstock G, Haap M, Hetzel J, Riessen R (2014). Nasal high-flow oxygen therapy in patients with hypoxic
respiratory failure effect on functional and subjective respiratory
parameters compared to conventional oxygen therapy and non-invasive
ventilation (NIV). BMC Anesthesiol.

[5] Stéphan F, Barrucand B, Petit P, Rézaiguia-Delclaux S, Médard A, Delannoy B, Cosserant B, Flicoteaux G, Imbert A, Pilorge C, Bérard L, BiPOP Study Group (2015). High-flow nasal oxygen vs noninvasive positive airway pressure in
hypoxemic patients after cardiothoracic surgery a randomized clinical
trial. JAMA.

[6] Frat JP, Thille AW, Mercat A, Girault C, Ragot S, Perbet S, Prat G, Boulain T, Morawiec E, Cottereau A, Devaquet J, Nseir S, Razazi K, Mira JP, Argaud L, Chakarian JC, Ricard JD, Wittebole X, Chevalier S, Herbland A, Fartoukh M, Constantin JM, Tonnelier JM, Pierrot M, Mathonnet A, Béduneau G, Delétage-Métreau C, Richard JC, Brochard L, Robert R, FLORALI Study GroupREVA Network (2015). High-flow oxygen through nasal cannula in acute hypoxemic
respiratory failure. N Engl J Med.

[7] Rittayamai N, Tscheikuna J, Rujiwit P (2014). High-flow nasal cannula versus conventional oxygen therapy after
endotracheal extubation a randomized crossover physiologic
study. Respir Care.

[8] Hernández G, Vaquero C, Colinas L, Cuena R, González P, Canabal A (2016). Effect of postextubation high-flow nasal cannula vs noninvasive
ventilation on reintubation and postextubation respiratory failure in
high-risk patients a randomized clinical trial. JAMA.

[9] Azevedo JR, Montenegro WS, Leitao AL, Silva MM, Prazeres JS, Maranhao JP (2015). High flow nasal cannula oxygen (HFNC) versus noninvasive positive
pressure ventilation (NIPPV) in acute hypoxemic respiratory failure A pilot
randomized controlled trial. Intensive Care Med Exp.

[10] Bell N, Hutchinson CL, Green TC, Rogan E, Bein KJ, Dinh MM (2015). Randomised control trial of humidified high flow nasal cannulae
versus standard oxygen in the emergency department. Emerg Med Australas.

[11] Brainard J, Scott BK, Sullivan BL, Fernandez-Bustamante A, Piccoli JR, Gebbink MG (2017). Heated humidified high-flow nasal cannula oxygen after thoracic
surgery A randomized prospective clinical pilot trial. J Crit Care.

[12] Parke RL, McGuinness SP, Eccleston ML (2011). A preliminary randomized controlled trial to assess effectiveness
of nasal high-flow oxygen in intensive care patients. Respir Care.

[13] Jones PG, Kamona S, Doran O, Sawtell F, Wilsher M (2016). Randomized controlled trial of humidified high-flow nasal oxygen
for acute respiratory distress in the emergency department the HOT-ER
study. Respir Care.

[14] Lemiale V, Mokart D, Mayaux J, Lambert J, Rabbat A, Demoule A (2015). The effects of a 2-h trial of high-flow oxygen by nasal cannula
versus Venturi mask in immunocompromised patients with hypoxemic acute
respiratory failure a multicenter randomized trial. Crit Care.

[15] Ansari BM, Hogan MP, Collier TJ, Baddeley RA, Scarci M, Coonar AS (2016). A randomized controlled trial of high flow nasal oxygen
(Optiflow) as part of an enhanced recovery program after lung resection
surgery. Ann Thorac Surg.

[16] Corley A, Bull T, Spooner AJ, Barnett AG, Fraser JF (2015). Direct extubation onto high-flow nasal cannulae post-cardiac
surgery versus standard treatment in patients with a BMI = 30 a randomised
controlled trial. Intensive Care Med.

[17] Futier E, Paugam-Burtz C, Constantin JM, Pereira B, Jaber S (2013). The OPERA trial - comparison of early nasal high flow oxygen
therapy with standard care for prevention of postoperative hypoxemia after
abdominal surgery study protocol for a multicenter randomized controlled
trial. Trials.

[18] Parke R, McGuinness S, Dixon R, Jull A (2013). Open-label, phase II study of routine high-flow nasal oxygen
therapy in cardiac surgical patients. Br J Anaesth.

[19] Fernandez R, Subira C, Frutos-Vivar F, Rialp G, Laborda C, Masclans JR (2017). High-flow nasal cannula to prevent postextubation respiratory
failure in high-risk non-hypercapnic patients a randomized multicenter
trial. Ann Intensive Care.

[20] Hernández G, Vaquero C, Gonzáles P, Subira C, Frutos-Vivar F, Rialp G (2016). Effect of postextubation high-flow nasal cannula vs conventional
oxygen therapy on reintubation in low-risk patients a randomized clinical
trial. JAMA.

[21] Maggiore SM, Idone FA, Vaschetto R, Festa R, Cataldo A, Antonicelli F (2014). Nasal high-flow versus Venturi mask oxygen therapy after
extubation Effects on oxygenation, comfort, and clinical
outcome. Am J Respir Crit Care Med.

[22] Mauri T, Turrini C, Eronia N, Grasselli G, Volta CA, Bellani G (2017). Physiologic effects of high-flow nasal cannula in acute hypoxemic
respiratory failure. Am J Respir Crit Care Med.

[23] Mauri T, Grasselli G, Jaber S (2017). Respiratory support after extubation noninvasive ventilation or
high-flow nasal cannula, as appropriate. Ann Intensive Care.

[24] Ni YN, Luo J, Yu H, Liu D, Ni Z, Cheng J (2017). Can high-flow nasal cannula reduce the rate of endotracheal
intubation in adult patients with acute respiratory failure compared with
conventional oxygen therapy and noninvasive positive pressure ventilation A
systematic review and meta-analysis. Chest.

[25] Zhao H, Wang H, Sun F, Lyu S, An Y (2017). High-flow nasal cannula oxygen therapy is superior to
conventional oxygen therapy but not to noninvasive mechanical ventilation on
intubation rate a systematic review and meta-analysis. Crit Care.

[26] Lin SM, Liu KX, Lin ZH, Lin PH (2017). Does high-flow nasal cannula oxygen improve outcome in acute
hypoxemic respiratory failure A systematic review and
meta-analysis. Respir Med.

[27] Furukawa TA, Watanabe N, Omori IM, Montori VM, Guyatt GH (2007). Association between unreported outcomes and effect size estimates
in Cochrane meta-analyses. JAMA.

[28] Lau J, Ioannidis JP, Terrin N, Schmid CH, Olkin I (2006). The case of the misleading funnel plot. BMJ.

[29] Macaskill P, Walter SD, Irwig L (2001). A comparison of methods to detect publication bias in
meta-analysis. Stat Med.

