# Supplementary material for: The effects of high-flow nasal cannula on intubation and re-intubation in critically ill patients: a systematic review, meta-analysis and trial sequential analysis
Source: Rev Bras Ter Intensiva. 2018 Oct-Dec;30(4):487–95. doi: 10.5935/0103-507X.20180070 (PMC6334477; doi:10.5935/0103-507X.20180070)
Supplement: Supplementary file 1 [file rbti-30-04-0487-suppl1.pdf]

# The effects of high-flow nasal cannula on intubation and re-intubation in critically ill patients: a systematic review, meta-analysis and trial sequential analysis

*Efeitos do uso de cateter nasal de alto fluxo na intubação e na reintubação de pacientes críticos: revisão sistemática, metanálise e análise de sequência de ensaios*

Rafael Ladeira Rosa Bocchile[1], Denise Carnieli Cazati[1], Karina Tavares Timenetsky[1], Ary Serpa Neto[1,2]

**Table 1S -** Subgroup analyses according to the strategy used in the control arm

| | Odds ratio (95%CI) | p value for interaction |
|---|---|---|
| Need for intubation | | 0.010 |
| *versus conventional oxygen therapy* | 0.54 (0.39 – 0.74) | |
| *versus NIV* | 0.98 (0.70 – 1.35) | |
| Need for therapy escalation | | 0.045 |
| *versus conventional oxygen therapy* | 0.66 (0.45 – 0.97) | |
| *versus NIV* | 0.98 (0.70 – 1.35) | |
| Mortality at the longest follow-up | | 0.990 |
| *versus conventional oxygen therapy* | 0.81 (0.56 – 1.19) | |
| *versus NIV* | 0.82 (0.40 – 1.69) | |
| Hospital mortality | | 0.770 |
| *versus conventional oxygen therapy* | 0.78 (0.52 – 1.16) | |
| *versus NIV* | 0.64 (0.18 – 2.26) | |

95%CI - 95% confidence interval; NIV - non-invasive ventilation.

**Table 2S -** Summary of findings and quality of evidence according to Grading of Recommendations, Assessment, Development and Evaluations (GRADE)

| Outcome | N of studies (participants) | Limitations | Inconsistency | Indirectness | Imprecision | Effect (OR) | Quality of the evidence |
|---|---|---|---|---|---|---|---|
| Need for intubation | 13 (3,555) | Moderate limitations | Serious inconsistency* | No serious indirectness | No serious imprecision | 0.72 (0.52 – 1.01) | Moderate |
| Therapy escalation | 15 (3,875) | Moderate limitations | Serious inconsistency* | No serious indirectness | No serious imprecision | 0.80 (0.59 – 1.08) | Moderate |
| Mortality at the longest follow-up | 9 (3,394) | Moderate limitations | Serious inconsistency* | No serious indirectness | No serious imprecision | 0.94 (0.70 – 1.25) | Moderate |
| Hospital mortality | 6 (2,119) | Moderate limitations | Serious inconsistency* | No serious indirectness | No serious imprecision | 0.84 (0.56 – 1.26) | Moderate |
| Need for NIV | 9 (1,454) | Moderate limitations | Serious inconsistency* | No serious indirectness | No serious imprecision | 0.64 (0.39 – 1.05) | Moderate |

OR - odds ratio; NIV - non-invasive ventilation. * Mild statistical heterogeneity

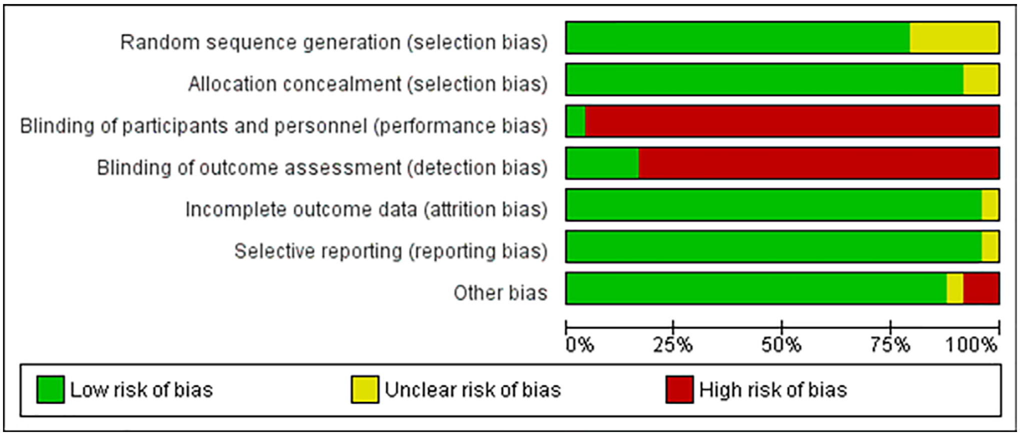


**Figure1S -** Global assessment of the risk of bias in the included studies.

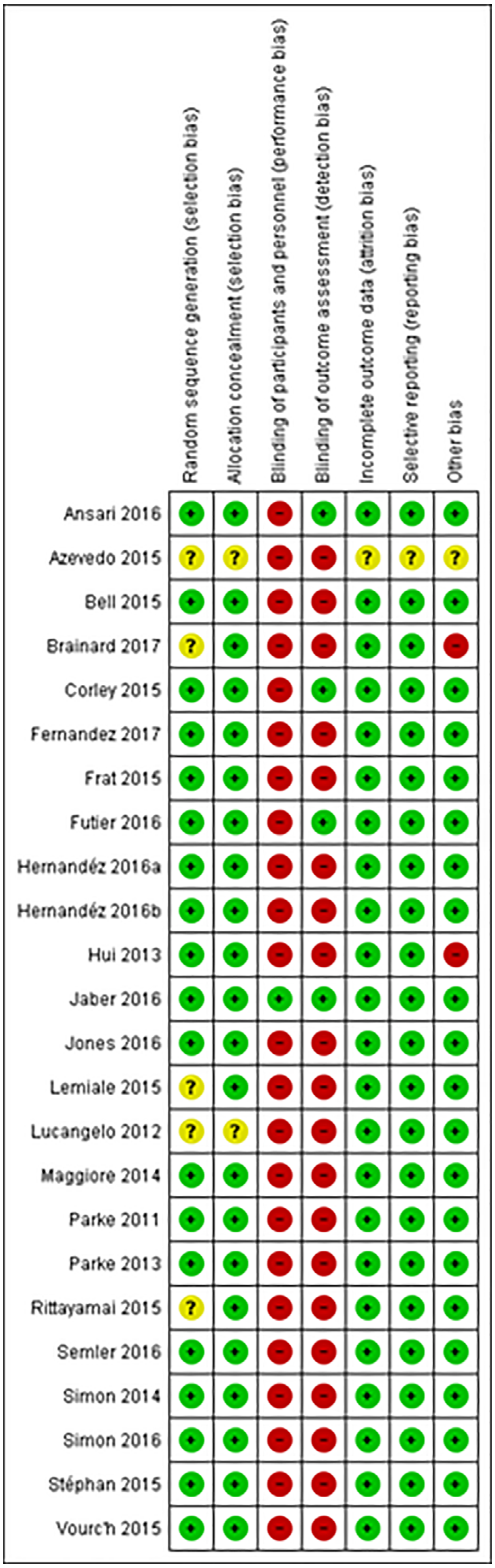


**Figure 2S -** Individual assessment of the risk of bias in the included studies.

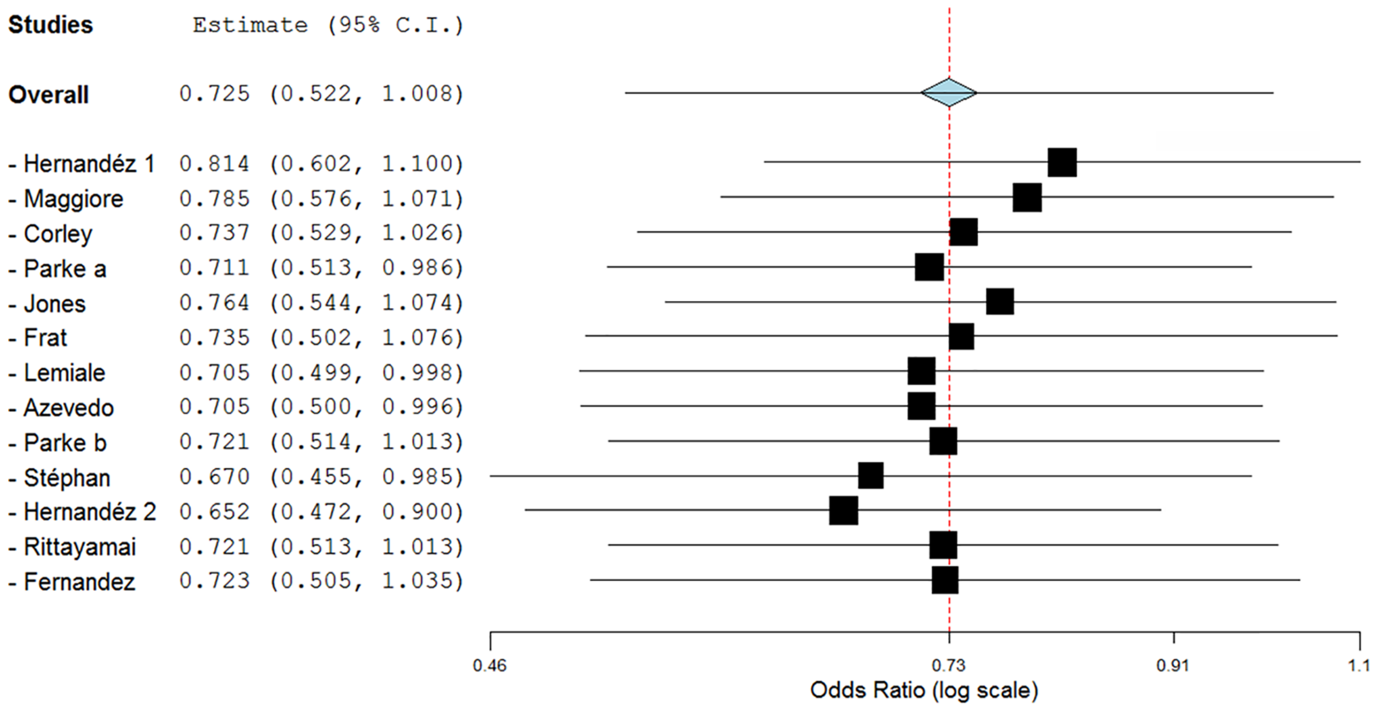


**Figure 3S -** "Leave-one-out" plot assessing the consistency of the findings for the primary outcome. 95%CI - 95% confidence interval.

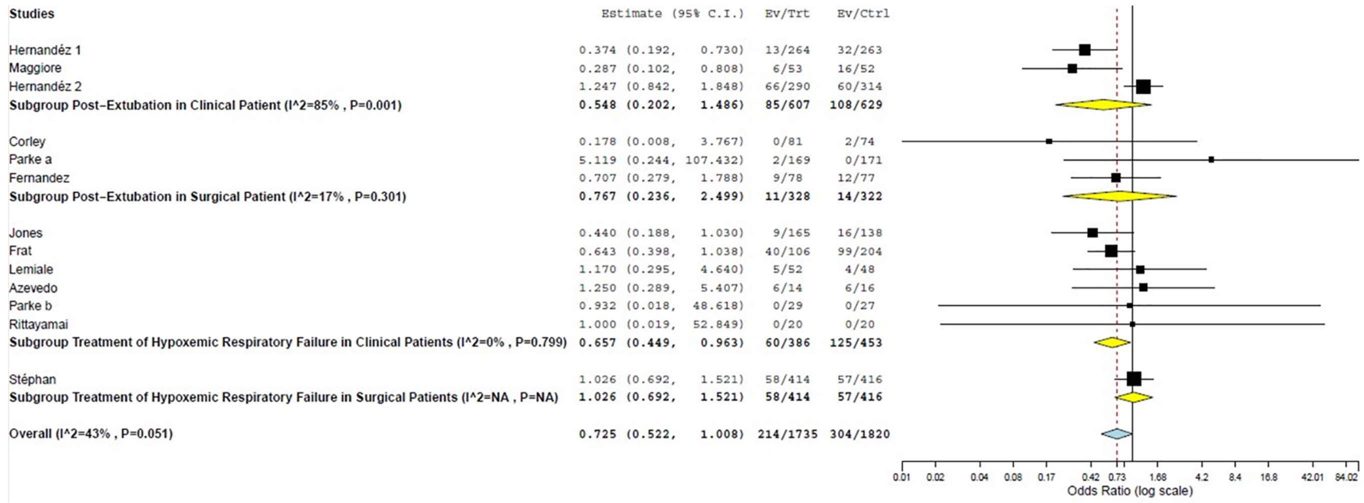


**Figure 4S -** Forest plot comparing the effects of high-flow nasal cannula with the control for the primary outcome (need for intubation or re-intubation) according to the indication. 95%CI - 95% confidence interval; EV - events; Trt - treatment; Ctrl - control.

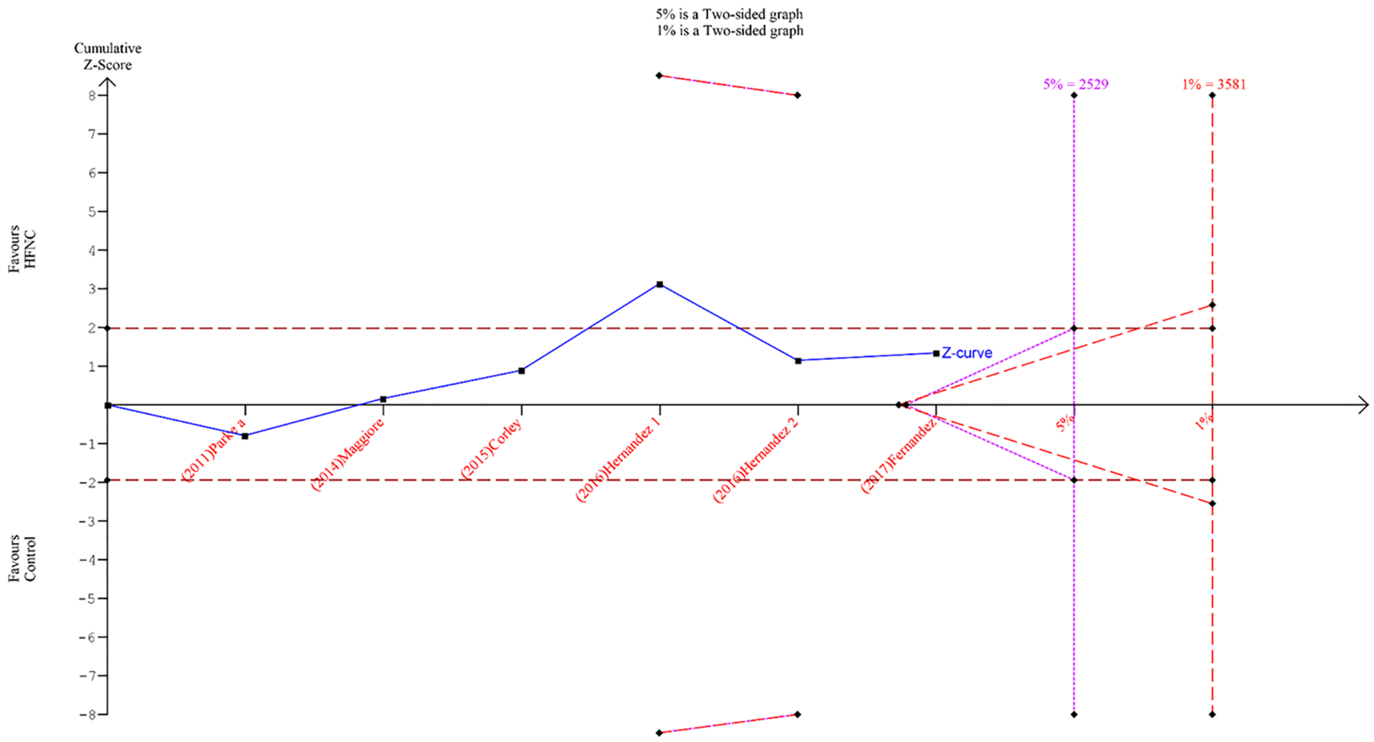


**Figure 5S -** Trial sequential analysis assessing the effect of high-flow nasal cannula in the primary outcome in the post-extubation subgroup. HFNC - high-flow nasal cannula. The cumulative meta-analysis (blue line) did not cross the efficacy boundary for the primary outcome (global type I error > 5%; purple line). The same was found when a more conservative boundary was used (red line).

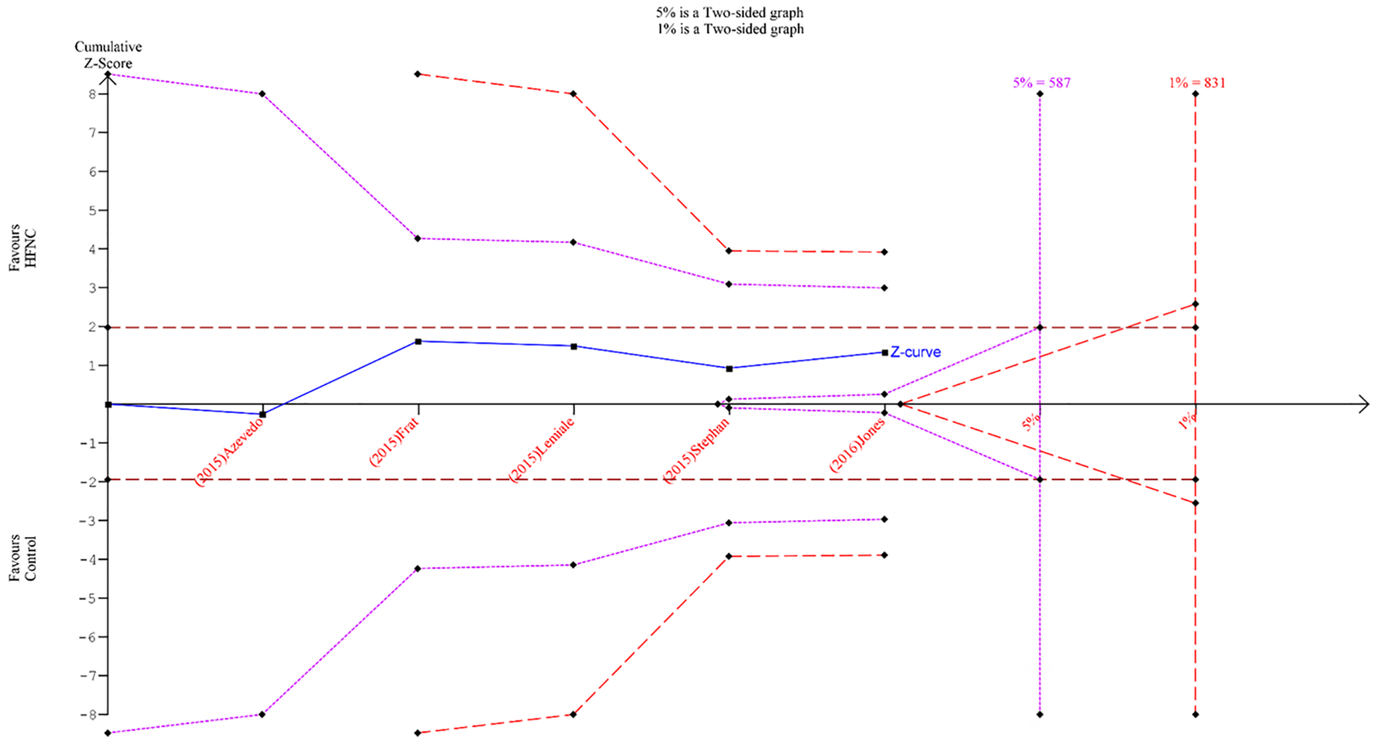


**Figure 6S -** Trial sequential analysis assessing the effect of high-flow nasal cannula in the primary outcome in the hypoxemic respiratory failure subgroup. HFNC - high-flow nasal cannula. The cumulative meta-analysis (blue line) did not cross the efficacy boundary for the primary outcome (global type I error > 5%; purple line). The same was found when a more conservative boundary was used (red line).
